# Identification of Novel Potential β-*N*-Acetyl-D-Hexosaminidase Inhibitors by Virtual Screening, Molecular Dynamics Simulation and MM-PBSA Calculations

**DOI:** 10.3390/ijms13044545

**Published:** 2012-04-10

**Authors:** Jianling Liu, Mengmeng Liu, Yao Yao, Jinan Wang, Yan Li, Guohui Li, Yonghua Wang

**Affiliations:** 1 College of Life Sciences, Northwest University, Xi’an, Shaanxi 710069, China; E-Mails: ljl2003ljl@126.com (J.L.); liuermengmeng@126.com (M.L.); 2 Center of Bioinformatics, Northwest A&F University, Yangling, Shaanxi 712100, China; E-Mails: liuermengmeng@gmail.com (Y.Y.); 7904059@126.com (J.W.); 3 School of Chemical Engineering, Dalian University of Technology, Dalian, 116012, China; E-Mail: adinalee@163.com (Y.L.); 4 Laboratory of Molecular Modeling and Design, State Key Laboratory of Molecular Reaction Dynamics, Dalian Institute of Chemical Physics, Chinese Academy of Sciences, Dalian 116023, China; E-Mail: ghli@dicp.ac.cn (G.L.); 5 State Key Laboratory of Crop Stress Biology for Arid Areas and College of Plant Protection, Northwest A&F University, Yangling 712100, China

**Keywords:** β-*N*-acetyl-d-hexosaminidase, OfHex1, inhibitor, virtual screening, molecular dynamics, MM/PBSA

## Abstract

Chitinolytic β-*N*-acetyl-d-hexosaminidases, as a class of chitin hydrolysis enzyme in insects, are a potential species-specific target for developing environmentally-friendly pesticides. Until now, pesticides targeting chitinolytic β-*N*-acetyl-d-hexosaminidase have not been developed. This study demonstrates a combination of different theoretical methods for investigating the key structural features of this enzyme responsible for pesticide inhibition, thus allowing for the discovery of novel small molecule inhibitors. Firstly, based on the currently reported crystal structure of this protein (OfHex1.pdb), we conducted a pre-screening of a drug-like compound database with 8 × 10^6^ compounds by using the expanded pesticide-likeness criteria, followed by docking-based screening, obtaining 5 top-ranked compounds with favorable docking conformation into OfHex1. Secondly, molecular docking and molecular dynamics simulations are performed for the five complexes and demonstrate that one main hydrophobic pocket formed by residues Trp424, Trp448 and Trp524, which is significant for stabilization of the ligand–receptor complex, and key residues Asp477 and Trp490, are respectively responsible for forming hydrogen-bonding and π–π stacking interactions with the ligands. Finally, the molecular mechanics Poisson–Boltzmann surface area (MM-PBSA) analysis indicates that van der Waals interactions are the main driving force for the inhibitor binding that agrees with the fact that the binding pocket of OfHex1 is mainly composed of hydrophobic residues. These results suggest that screening the ZINC database can maximize the identification of potential OfHex1 inhibitors and the computational protocol will be valuable for screening potential inhibitors of the binding mode, which is useful for the future rational design of novel, potent OfHex1-specific pesticides.

## 1. Introduction

Broad-spectrum neuroactive pesticides have been used for decades as the major instrument for controlling arthropod pests in agriculture, forestry, stored-products, and in public health. The massive and widespread use of such nonselective pest control agents have caused many serious toxicological and environmental problems that are amply recorded [1]. Furthermore, the increasing awareness of the public to hazards associated with toxic residues in drinking water and edible crops has become a considerable stimulus to design alternative pest control measures, including more selective pesticides. Among the selective, targets only the enzymes involved in chitin degradation have long been regarded as species-specific target potentials in developing environmentally-friendly pesticides.

Chitin, a highly insoluble biopolymer, is a straight chain homopolymer composed of β-1,4 linked *N*-acetyl-d-glucosamine monomers (GlcNAc), and is distributed widely in nature. It is a major component of the exoskeletal structure of arthropods, including crustaceans and insects, as well as mollusks, nematodes, and worms. More specifically, chitin is found in the cuticle of the integument and peritrophic membrane of the midgut. The cuticle provides support and protection through its rigidity, and prevents water loss from the body surface. In gut tissues, a cuticular layer also lines both the foregut and hindgut. The peritrophic membrane of the midgut, one function of which is to protect cells lining the midgut from abrasion by food particles, also contains chitin. The insect undergoes periodic shedding or molting of the cuticle to allow for growth and maturation [2]. During the molting, fluid that contains hydrolytic enzymes to degrade chitin and protein is secreted in the cuticle. Therefore, any interference with chitin deposition or its untimely degradation is detrimental to the insect involved. Two hydrolytic enzymes are involved in the sequential degradation of chitin, *i.e.*, chitinase (EC3.2.1.14), and *N*-acetylglucosaminidase (EC 3.2.1.30) [3]. In insect, chitinase firstly hydrolyzes the polysaccharide chitin into GlcNAcoligomers, and which is further hydrolyzed into *N*-acetyl-d-glucosamine (GlcNAc) by β-*N*-acetyl-d-hexosaminidase (EC3.2.1.52) [4]. The ratio of these two enzymes regulates the efficiency of chitin degradation and thus affects remodeling of the chitin components of insects [5]. Because plants, vertebrates and humans were thought not to possess or process chitin, the enzymes involved in chitin hydrolysis have been considered as potential specific and safe targets for the development of environmentally-friendly pesticides [1].

β-*N*-acetyl-d-hexosaminidases are expressed in different organisms and also play various physiological roles in the specific life cycles. In plants, β-*N*-acetyl-d-hexosaminidases regulates the post-translational modification of *N*-glycan [6] and might participate in defense-related processes [7]. In mammals, β-*N*-acetyl-d-hexosaminidases catalyze the hydrolysis of terminal, non-reducing *N*-acetyl-d-glucosamine (GlcNAc) and *N*-acetyl-d-galactosamine (GalNAc) residues in glycoproteins, gan-gliosides, and glycosaminoglycans [8]. Chitinolytic β-*N*-acetyl-d-hexosaminidases are only involved in insect chitin degradation processes, which is different from chitinases, which exist in a wide range of species from viruses, to plants, and animals, thus the selective inhibitors targeting this particular enzyme might be not harmful to plants and mammals. This explains why the commercial chitinase insecticides, allosamidin [9], argifin [10] and argadin [11] for example, have broad inhibitory activities towards various organism [12]. Recently, an insect β-*N*-acetyl-d-hexosaminidase, *i.e.*, OfHex1, has been reported from the Asian corn borer *Ostrinia furnacalis* (Guenée) [13] and has been shown to play a vital role during the pupation of *O. furnacalis*. This provides a new chance to develop novel pesticides with species specificity and selectivity. However, to our knowledge, no OfHex1 inhibitors have been reported until now.

Searching for a proper lead compound for a given molecular target is a critical step in the drug discovery process. Traditionally, high-throughput screening (HTS) of large chemical libraries has been a primary source of identification of novel lead compounds. Nowadays, virtual screening is becoming increasingly one of the most powerful computational tools for the rapid discovery of novel and original chemical entities with potential activity [14,15]. These techniques are now also used to help understand the binding mode of active compounds and identify new hits. So far, application of a docking-based virtual screening approach, with the crystal structure of β-*N*-acetyl-d-hexosaminidase, has not been published. In order to find novel OfHex1 small molecule inhibitors with diverse chemical scaffolds, in this work, we have carried out a virtual screening process of ZINC [16] based on a set of in-house developed criteria. The selected lead-like compounds were subjected to a multistep docking-based simulation with the crystal structure of OfHex1 (3NSN.pdb). The more promising compounds were further calculated by Molecular Dynamics (MD) simulations and the binding free energy were calculated by using the molecular mechanics/Poisson Boltzmann surface area (MM/PBSA) methodology [17]. For the first time, this work performs high throughput virtual screening on the ZINC database to maximize the identification of potential OfHex1 inhibitors and to use MD simulation to investigate protein–ligand binding stabilities, which might be helpful for understanding the interactions between this enzyme with its potential inhibitors and also be useful in the design of novel environmentally-friendly insecticides.

## 2. Results and Discussion

**Figure 1:**
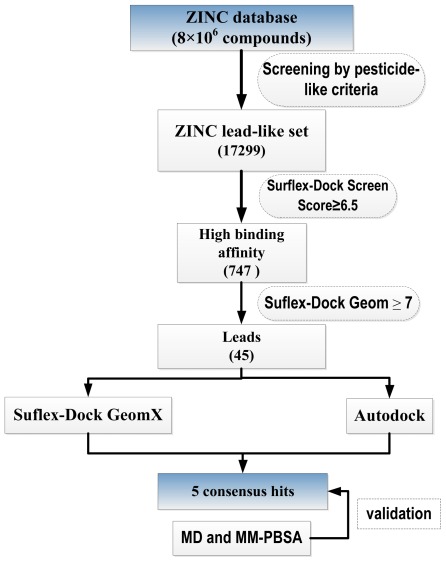
Flowchart of the multistep virtual screening strategy implemented in this work. After selecting lead-like compounds, the ZINC Database was subsequently filtered with Surflex-Dock screen and Surflex-Dock Geom. Top-ranked compounds were docked with Surflex-Dock GeomX and AUTODOCK. Consensus hits were identified and Molecular dynamics simulation, MM-PBSA calculations were carried out for validation.

The main focus of a virtual screening procedure is to reduce the size of a chemical library, providing a focused subset of molecules, enriched in compounds likely to be active. The general working process of the multistep docking approach used in this job was presented in Figure 1. After selecting lead-like compounds, we employed a fast docking protocol to further filter the ZINC lead-like set. As part of the screening, two programs were used to screen the database sequentially. Top scored hits with favorable docking conformation were selected and then the MD simulation studies and molecular mechanics Poisson–Boltzmann surface area (MM-PBSA) binding free energy calculations for these selected docking complexes were performed. Details of the virtual screening approach, MD simulations and the binding free energy calculations of the selected docking complexes are presented below.

### 2.1. Virtual Screening

Virtual screening is a proficient approach in discovering inhibitors with novel chemical scaffolds. In the present case, 17,299 compounds were screened out of the ZINC database, based on expanded pesticide-likeness criteria.

**Figure 2:**
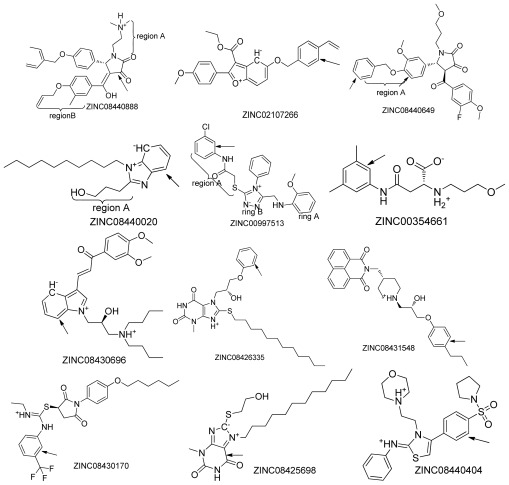

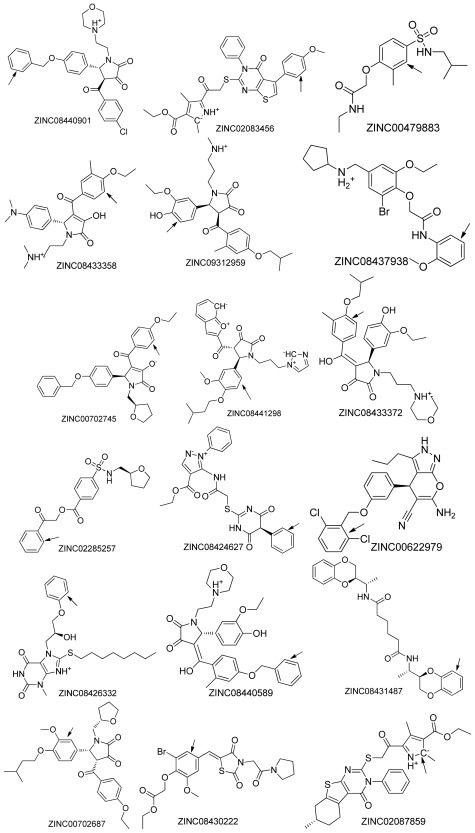

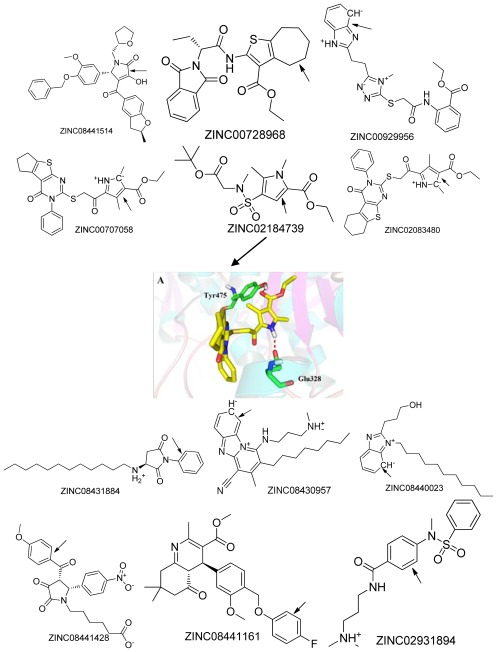
Chemical structures of the 45 top scoring compounds. Top scoring compounds with poor binding mode inside the active site of the protein (pdb id: 3NSN) are indicated with arrows.

By Surflex-Dock Screen program, 747 molecules with docking score ≥ 6.5 were selected, which were further refined by the Surflex-Dock Geom, resulting in 45 unique compounds with Suflex-Dock Geom score ≥ 7.0. All these obtained compounds were further independently docked with AutoDock and Suflex-Dock GeomX, respectively. The docking scores, docking binding energy, selected 45 compounds properties and chemical structures are presented in Table 1 and Figure 2, respectively. Among the Surflex-Docking consensus hits, a total of 27 compounds with AutoDock Energy ≤ −8.0 kcal/mol and Surflex-Dock GeomX score ≥ 8 were identified. This set can be regarded as Surflex-Dock-AutoDock hits set and the molecular properties and chemical structures of this hit set were described in Table 1 (the first 27 compounds) and Figure 2, respectively.

### 2.2. Top Scoring Compounds

Table 1 shows all the obtained 45 compounds with their AutoDock binding energy ≤ −5 kcal/mol and Surflex scores ≥ 8, which indicated that these molecules have good binding affinities with OfHex1. Interestingly, all compounds have an N^+^ atom (ZINC08430957) or an H^+^ atom (ZINC08440404) in the structure as shown in Figure 2. Notably, the docking analysis revealed that most compounds share similar binding patterns in the active pocket of OfHex1, with the terminal aromatic rings (indicated by arrow in Figure 2) oriented toward the binding center.

As evident from the virtual screening, all these 45 hits performed well with a Surflex docking score. However, some of the hits were represented with an unfavorable Autodock binding energy value (8.8 and −5.53 kcal/mol for ZINC08441428). Therefore, the 27 compounds which performed well both in terms of Autodock binding energy values (bind energy ≤ −8.0 kcal/mol) and docking scores (Surflex score ≥ 8.0) were selected for the final docking complexes analysis.

It has been proven that two residues Trp448 and Trp490 were essential for the binding of inhibitor TMG-chitotriomycin of enzyme OfHex1, and the mutation of these two residues would cause a loss of more than 2000-fold activity for this enzyme [18]. Currently, although the binding mode of OfHex1 with its inhibitors still remained unclear, we hypothesize these two residues might be involved in recognition of inhibitors. Therefore, all the docking conformations of each compound have been checked and only those ligands which interact with these two key residues by π–π interactions or hydrogen-bonding interactions and which also possess high binding scores (Surflex score ≥ 8.5) and low binding energy (bind energy ≤ −8.5 kcal/mol) were kept for further analysis. Consequently, 5 compounds (the first five compounds in Table 1 and Figure 2) from the total 27 compounds with the π–π or hydrogen-bonding interactions with residues Trp448 and Trp490 were obtained. An example of a Surflex-Dock-AutoDock hit (Compound ZINC02083480) with poor docking mode is represented in Figure 2A.

### 2.3. Molecular Dynamics Simulation

**Figure 3:**
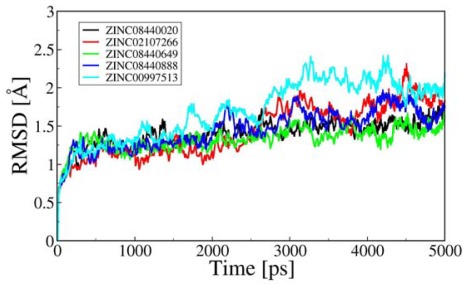
Root-mean-square deviations (RMSD) of all Cα atoms of the complexes during the production phases relative to the initial structures. (black line, ZINC08440020-OfHex1; red line, ZINC02107266-OfHex1; green line, ZINC08440649-OfHex1; blue line, ZINC08440888-OfHex1; light blue line, ZINC00997513-OfHex1.

A 5 ns MD simulation followed by molecular docking was performed for the five complexes. The root-mean-square deviation (RMSD) values of the ligands in all simulations, compared to their starting points, were monitored and only those simulations in which the ligand exhibited a stable binding position were considered for further analysis. A stable binding position was defined by the ligand still being within 2.5 Å RMSD of all other stable ligand placements in its group at the end of the simulation. RMSD values of the protease C_α_ atoms relative to the initial structure were calculated and plotted in Figure 3. In the first 500 ps, the RMSD values increased quickly, which means that the structures of the five OfHex1-inhibitor complexes dissolved in the solution relax and remove the repulsion within the systems. As seen from Figure 3, ZINC08440649 and ZINC08440020 systems have reached the equilibrium after 2.5 ns of MD simulations with a RMSD value around at 1.54 Å while ZINC02107266, ZINC00997513 and ZINC08440888 systems have reached the equilibrium after 3 ns, and RMSD values of the ZINC02107266 and ZINC08440888 complexes were fluctuating around 1.78, 1.95 and 1.72 Å, respectively. The results suggested that the stabilities of the dynamic equilibriums for the five complexes are reliable and the trajectories can be applied to collect snapshots for further analyses. In addition, the stability of the system also proved the credibility of the docking results.

Thus, the average structures of the five complexes were obtained from the last 1 ns on the MD trajectory and were carefully analyzed to explore the binding mechanism of OfHex1 with its potential inhibitors.

### 2.4. Binding Mode

**Figure 4:**
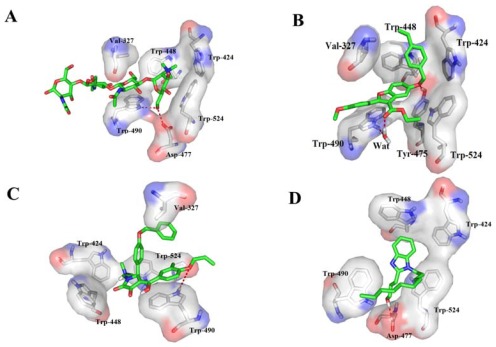

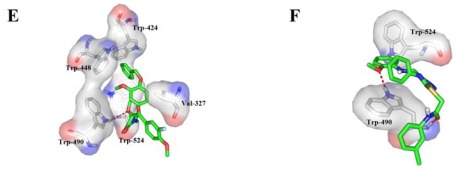
(**A**) Docked conform at ions of OfHex1 crystal structure (magenta) with active compound TMG-chitotriomycin(green); (**B**) Binding interactions of compound ZINC02107266 at the binding site of the OfHex1 receptor; (**C**) Binding interactions of compound ZINC08440888 at the binding site of the OfHex1 receptor; (**D**) Binding interactions of compound ZINC08440020 at the binding site of the OfHex1 receptor; (**E**) Binding interactions of compound ZINC08440649 at the binding site of the OfHex1 receptor; (**F**) Binding interactions of compound ZINC00997513 at the binding site of the OfHex1 receptor.

The correct mode of ligand-protein binding is extremely important not only for molecular recognition but also for its implication in drug discovery. However, the detailed mechanism for OfHex1-ligand interactions still remained unclear. As a general rule, in most of the proteinases where the inhibition of the enzyme is brought about by inhibitor ligands, specific hydrogen bond and hydrophobic interactions between the inhibitors and the active site subsites of the enzyme have been found to be responsible for mediating protease inhibition [19]. Taking into consideration of these factors, we mainly focused on hydrogen bond and hydrophobic interactions between ligands and receptors. The average 3D-structures of complexes were compared with the X-ray crystallographic structure of 3NSN (Figure 4A–F). It was shown that these five compounds binding in the active site share broadly similar conformation with TMG-chitotriomycin presented in 3NSN.

As shown in the Figure 4B–F, after a 5 ns MD simulation all the 5 compounds obtained almost the same binding mode as the TMG-chitotriomycin in 3NSN (Figure 4A) which indicated that these compounds bind to the OfHex1 pocket in a favorable conformation. The hydrophobic pocket is mainly formed by three tryptophan residues (Trp424, Trp448 and Trp524), with Asp367 and Tyr475 forming the wall and bottom, and the Trp490 and Val327 producing the entrance of the cavity. The key residues involved Trp424, Trp448, Trp524, Trp490 and Val327 which offered a hydrophobic interaction for the compounds (Figure 4B–F). The benzene groups of the potential inhibitors were bounded by a hydrophobic pocket consisting of residues such as Trp424, Trp448, Trp524, Trp490 and Val327 except the compound ZINC00997513 which only formed hydrophobic interaction with Trp524 and Trp490. The methylene carbons and the benzene rings of the ligands also produce strong hydrophobic interactions with residues Trp490 and Val327 could stabilize the complex. These observed hydrophobic interactions were significant for stabilization of the ligand–receptor complex. In addition, the ligands were also sandwiched by Val327 and Trp490, through π–π stacking or hydrogen-bonding interactions with residue Trp490. Although these five compounds produced a major cluster of binding conformations overlapping a hydrophobic binding region, it was interesting to note that each compound also formed specific hydrogen bonding interactions with the enzyme. For compound ZINC02107266 (Figure 4B), the ketone from the region A accepted a hydrogen-bond (–O···H–N–, 2.82 Å) to Tyr475 and the oxygen atom formed a hydrogen-bond with Trp490 via a water molecule. Clearly, these additional interactions would be favorable for the binding energy (*E*_b_ = −10.2 kcal/mol) and total docking score (9.03). The N^+^ of the region A of ZINC08440888 produced a strong arene-cation interaction with Trp424 and Trp448, respectively. In addition, the oxygen atom from the region B also formed H-bond with Trp490, with the benzene ring undergoing a π–π stacking interaction with Trp490 (Figure 4C). For ZINC08440020, the benzene and purine rings made hydrophobic interactions with Trp424 and Trp448, respectively (Figure 4D). The oxygen atom of region A H-bonded to Asp477 (–O–H···O–, 2.7 Å), which was also crucial for stabilizing the inhibitor in TMG-chitotriomycin (3NSN.pdb). Compound ZINC08440649 penetrated into the hydrophobic pocket and formed H-bonding with Trp490(–O···H–N–, 3.1Å) (Figure 4E). For compound ZINC00997513, The benzene ring in region A made hydrophobic contact with Trp524 and Trp490, with the ketone group H-bonded to Trp490 (–O···H–N–, 2.97Å) and the oxygen atom to Trp490 (–O···H–N–, 2.41Å). In addition, the ring B also formed a π–π stacking interaction with Trp490 (Figure 4F).

Based on all these results, we proposed that the cavity consisted of Trp424, Trp448, and Trp524 led to an increase in the net hydrophobicity of the active pocket and this was significant for the orientation of the inhibitors within the active site of OfHex1, the hydrogen-bonding and π–π stacking interactions with residues Asp477, Tyr475 and Trp490 could increase the inhibitor binding affinity to the enzyme, and also the hydrophobic interactions with residues Trp490 and Val327 could stabilize the complex and block the entrance of the active pocket of OfHex1.

### 2.5. Binding Free Energy Calculation

Among the several solvation models, PBSA is regarded as an attractive approach for drug design since it works well not only for small and medium size organic compounds but also for the biological molecules such as proteins and DNA [20]. In MM-PBSA calculations, the affinity of the ligand binding to the protein can be estimated using the snapshots from a trajectory of the complex (the single-trajectory protocol) [21].

The total binding free energy, Δ*G*_bind_, for the five OfHex1-inhibitor complexes were summarized in Table 2. To further understand major determinants for the binding of inhibitors to the protein, the binding free energy of each inhibitor was decomposed to the contributions including van der Waals energy, electrostatic energy, solvation free energy, polar solvation energy and entropy (−*T*Δ*S*). As it was shown in Table 2, both van der Waals and electrostatic terms in the gas phase interactions with the protein dominated the binding process, whereas the electrostatic component of the solvation free energy (Δ*G*_PB_ > 0) and the entropy terms (−*T*Δ*S*) impaired the binding process. The nonpolar solvation energies (Δ*G*_np_), which correspond to the burial of SASA upon binding, slightly contributed to the inhibitors binding, particularly for the ZINC08440649, ZINC08440020, ZINC02107266 and ZINC00997513 complexes, it is obvious that the van der Waals interactions were a major contributor to the binding of inhibitors. The values of the van der Waals contribution of the four complexes were −40.84 kcal/mol, −41.12 kcal/mol, −32.56 kcal/mol, and −31.81 kcal/mol, respectively. This was in line with the fact that the binding pocket of the receptor is mainly composed of hydrophobic residues such as Trp424, Trp448, Trp524, Trp490, Val327. The calculated electrostatic interaction contributions to the binding free energies for all of the complexes, except for ZINC08440888 and ZINC02107266, were also very close to each other, ranging from −20.28 to −28.59 kcal/mol. This was because the hydrogen-bonding interactions in the three complexes (with ZINC00997513, ZINC08440649, ZINC08440020) were similar which were also consistent with the fact that these hydrogen-bonding interactions found to be significant for the stabilization of the inhibitor TMG-chitotriomycin (3NSN.pdb). For the complex with ZINC08440888, the electrostatic interaction contribution (−42.52 kcal/mol) is significantly more favorable. This might be due to the fact that compound ZINC08440888 has an N^+^ atom in the region A that increases the electrostatic term in the binding free energy. For the complex with ZINC02107266, the electrostatic interaction (−5.99 kcal/mol) slightly contributed to the inhibitors binding as compared with other four complexes. This large difference might reflect that a hydrogen bond between the hydroxyl of Tyr475 and the carbonyl functionality of the ZINC02107266 were not essentially stabilizing interaction to those hydrogen bond interactions with other residues observed in other four complexes. Therefore, both the van der Waals and the electrostatic contributions terms in the gas phase played key roles in the activities of these compounds. Overall, the relative binding free energy of the ZINC08440649, ZINC08440888, ZINC02107266 and ZINC08440020, ZINC00997513 complexes were −20.55 kcal/mol, −28.37 kcal/mol, −16.92 kcal/mol, and −19.34 kcal/mol, −19.23 kcal/mol, respectively. This indicated that these five compounds had a good binding free energy with OfHex1, the five docking complexes were expected to be stable and the binding mode of these five complexes were indicated to be reliable.

## 3. Materials and Methods

### 3.1. Lead-like Selection

A collection of “drug-like” molecules with more than 8 × 10^6^ compounds was obtained from the ZINC database [22], which includes a wide range of chemical structures. In order to further select a subset of lead-like compounds, the original set of 8 × 10^6^ entries has been reduced to 23,891 compounds by applying the expanded pesticide-likeness criteria [23]: molecular weight between 150 and 800 Da; the presence of 0–3 hydrogen-bond acceptors and 1–10 hydrogen-bond donors; and overall hydrophobicity 0 < xlog *P* ≤ 7.0; polar surface area (PSA) ≤ 300 Å^2^. The obtained database, contained 24,904 compounds and we removed 7605 compounds with the same SMILES. For docking, a total of 17,299 molecules (referred in this work as the lead-like set) with unique SMILES were selected.

### 3.2. Docking with Surflex-Dock

The crystal structure of β-*N*-acetyl-d-hexosaminidase (OfHex1, 2.1 Å, 3NSN.pdb) was selected as the docking template. Prior to docking studies, crystallographic water molecules in the structure were removed, hydrogen atoms were added to the protein structure, all atom force field charges and atom types were assigned with using the SYBYL × 1.1 software.

After the protein preparation, the virtual screening process was performed by the Surflex docking (the v 2.51 module in SYBYL × 1.1 package), a virtual screening tool that combines Hammerhead’s empirical scoring function [24] with morphological similarity to generate putative poses of ligand fragments. Surflex provide three different levels of docking precision (Surflex-Dock screen, high-throughput virtual screening; Surflex-Dock Geom, standard precision; Surflex-Dock GeomX, extra precision). We carried out our calculations in Surflex-Dock screen first, and then Surflex-Dock Geom mode. The Surflex program works through (1) generation of an active site (called protomol), the two factors “proto_thresh” was set to 0.5 and “proto_bloat” was left at the default (0); (2) Fragmentation of each of the individual ligands are then aligned to the protomol so as to yield poses that maximize molecular complementarity with the binding site; (3) A full molecule is then positioned from the aligned fragments and scored using an empirically derived function including charged and hydrogen bond polar terms, solvation, entropic, and hydrophobic complementarity terms; (4) The top ranked molecules were further validated by AutoDock and Surflex-Dock GeomX mode, respectively.

### 3.3. Docking with AUTODOCK

AutoDock tools (ADT) (version 1.4.5) [25] were used for protein and ligand preparation. Briefly for protein, all hydrogen, including non-polar, Kollman charges, and solvation parameters were added to all atoms. After adding charges, the non-polar hydrogens were merged [26]. The auxiliary program Autogrid was used to generate the grid maps; where each map was centered at the coordinates of docked TMG-chitotriomycin with the model of OfHex1 as recently reported [18]. The docking area was defined using AutoGrid 4.2.3. A 60 × 60× 60 3-D affinity grid centered around the TMG-chitotriomycin binding site [18] with a 0.375 Å grid point space was identified. For all ligands, Gasteiger charges [27] were assigned and then nonpolar hydrogen were merged. All bond rotations for ligands were ignored and the Lamarckian genetic algorithm (LGA) [28] was employed for ligand conformational searching because it has enhanced performance relative to simulated annealing or the simple genetic algorithm and then an AutoDock 4.2.3 protocol was applied. For each compound, we used the default docking parameters with the exception of the followings: initial population of 100 randomly placed individuals, maximum number of 2.5 × 10^6^ energy evaluations and maximum number of 2.7 × 10^4^ generations. The mutation rate and crossover rate were set to 0.02 and 0.80, respectively. The elitism value was set to 1 and the local search frequency to 0.06. Ten independent docking runs were carried out for each ligand using these parameters for rapid screening. The best docked position was determined by comparing docking poses and considering the total energy value. Among several similar docking poses, the more energetically favorable conformation was selected.

### 3.4. Molecular Dynamics (MD) Simulations

To take the protein flexibility into account in the docking process and estimate the binding free energies of the complexes, we used the MD computational approach. The MD simulations on the five complexes were carried out by using the AMBER 10 suite of programs [29]. The general AMBER force field (GAFF) was chosen for the ligands [30] and the standard AMBER force field for bio-organic systems (ff99SB) was chosen to describe the protein [31]. All the systems were immersed in a truncated octahedral box of water molecules (TIP3P) with a margin of 10 Å along each dimension and counterions were added to neutralize the system. Then the cut-off distance was kept to 8 Å to compute the nonbonded interactions. All simulations were performed under periodic boundary conditions [32], and long-range electrostatics were treated by using the Particle-mesh-Ewald method (PME) [33]. The SHAKE algorithm [34] was applied to all bonds.

Prior to MD simulation, the systems were minimized by 200 steps of steepest descent followed by 800 steps of conjugate gradient to remove the bad contacts in the structure. The first step of MD simulations was that the systems were gradually heated to 300 K at a constant force of 2.0 kcal/mol·Å^−2^ constraining the protein atoms. The second step consisted of a 50 ps pressure-constant period to raise the density while still keeping the complex atoms constrained. The third step was a 500 ps Langevin dynamics with a collision frequency of 1.0 ps^−1^, which was applied to control the temperature of the system. Finally, a 5 ns dynamics simulation without restriction was performed at a constant temperature of 300 K and a constant pressure of 1 atm. During the sampling process, coordinates were saved every 2 ps in the simulations of the complexes.

### 3.5. MM/PBSA Calculations

Binding free energies were calculated using the Molecular Mechanics-Poisson Boltzmann Surface Area (MM-PBSA) method [35] integrated in Amber 10 [29]. We extracted the last 500 snapshots, taken at 2 ps intervals from the trajectories of each simulation for the energy calculation. The interaction energy was calculated according to the following equation:

(1) $$\Delta G=\Delta E_{MM}+\Delta G_{sol}^{polar}+\Delta G_{sol}^{nonpolar}-T\Delta S_{solute}$$

where Δ*E*_MM_ is the gas-phase energy, denoting the sum of molecular mechanical (MM) energies of molecules from internal (Δ*E*_int_), electrostatic (Δ*E*_ele_), and van der Waals energies (Δ*E*_vdw_). The solvation free energy (Δ*G*_sol_) is composed of polar (Δ*G*_sol polar_) and nonpolar (Δ*G*_sol nonpolar_) parts. *T*Δ*S* is the contribution of conformational entropy to the binding. Here, the polar solvation free energy was calculated by solving the Poisson-Boltzmann equation using the program Delphi II [36]. The dielectric boundary was defined using a 1.4 Å probe on the atomic surface. The values of the interior dielectric constant and the exterior dielectric constant were set to 2 and 80, respectively. The non-polar solvation free energy was calculated from the solvent-accessible surface area (*SASA*) algorithm:

(2) $$\Delta G_{nonpolar}=\gamma SASA+b$$

*γ* is the surface tension proportionality constant (the value is 0.00542 kcal/mol Å^2^). The free energy of nonpolar solvation for a point solute (*b*) is set to 0.92 kcal/mol.

During the process of formation of a protein-ligand complex, the vibrational entropy of the ligand and the torsional motion of the protein were restricted. The total entropy term was repulsive, opposed the binding, since the ligand lost three degrees of rotations and vibrations upon binding, and these degrees were converted to the vibrational motions. The contribution of the entropy change (*T*Δ*S*) to the binding free energy arises from changes in the translational, rotational and vibrational degrees of freedom. Because the contributions from translation and rotation are much smaller than vibration, *T*Δ*S* is calculated using normal-mode analysis using the NMODE module in AMBER 10 [37]. 2Because entropy calculations for large systems are extremely time consuming; only 10 snapshots were taken at an interval of 20 ps from the final 1 ns of the MD simulation to calculate the entropy contribution.

## 4. Conclusions

We reported on a systematic computational screening of a large collection of compounds as OfHex1 potential inhibitors. To the best of our knowledge, this is the first *in silico* study of ZINC database towards the identification of novel compounds with potential inhibitory activity on OfHex1. In addition, we also performed molecular docking and molecular dynamic simulations to explore the potential binding mechanisms for OfHex1 inhibitors. In our proposed binding mode, we found that the binding site consists of one main hydrophobic pocket, which leverages the steric interference between the enzyme and hydrophobic region such as benzene groups in the ligands. The MD validated results further demonstrated that the hydrophobic interactions for enzyme and ligands were mainly produced by Trp424, Trp448, Trp524, Val327 and Trp490, which was in agreement with the previous crystallographic results. From the analysis of five complexes, the benzene rings were mainly captured by Trp424, Trp448 and Trp524 for four potential inhibitors (ZINC08440649 ZINC02107266 ZINC08440020 and ZINC00997513), while the N^+^ of ZINC08440888 produced a strong arene-cation interaction with Trp424 and Trp448. This further demonstrated that N^+^ and the benzene ring are important in the selective recognition of ligand-receptor.

In addition, our MD results also showed that the π–π stacking interaction with Trp490 and the inter-hydrogen bonding with Asp477 and Tyr475 are important for ligand stabilization in the receptor. The MM-PBSA results also demonstrated that the five obtained potential inhibitors exhibited good binding affinity with OfHex1. The obtained results might be helpful for the development of novel, potent OfHex1-specific pesticides.

## Figures and Tables

**Table 1:** Calculated lead-like related properties, docking energy and docking scores of representative virtual screening hits.

NO.	Compound (ZINC ID)	MW a	xLogP a	HBA a	HBD a	PSA a	Autodock4 Docking energy b	Surflex score b
1	08440649	535	5.07	8	0	102	−8.65	9.06
2	08440888	527	6.28	4	1	120	−9.78	10.86
3	02107266	428	7	5	0	63	−10.2	9.03
4	08440020	316	5.68	1	1	50	−8.66	10.31
5	00997513	479	4.39	4	2	105	−8.52	8.96
6	02931894	376	2.7	1	0	119	−10.42	8.6
7	08440404	500	0	1	2	110	−10.02	9.68
8	08431884	359	7	2	2	93	−9.55	11.6
9	08440901	534	5.9	5	1	134	−9.35	8.83
10	08430957	420	6.2	0	2	86	−9.12	10.15
11	08437938	478	4.3	0	3	86	−9.02	10.9
12	01823357	342	5	0	3	96	−8.7	10.1
13	08431492	468	3	0	2	60	−8.7	9.9
14	08430170	522	7	7	2	97	−8.69	9.86
15	08424627	491	3.1	0	2	200	−8.65	10.3
16	02728968	454	5.9	2	1	107	−8.65	9.3
17	02087859	535	7.3	0	0	156	−8.6	9.7
18	08430696	493	6.76	4	2	99	−8.54	11.03
19	08425698	410	5.3	2	2	158	−8.53	9.29
20	02083480	521	6.7	2	1	149	−8.3	8.9
21	08441543	533	6.2	0	0	126	−8.23	9.9
22	00622979	455	7	0	3	194	−8.2	10.3
23	08441161	479	5.3	1	0	134	−8.2	8.7
24	08440023	330	6.2	2	1	51	−8.19	9.76
25	00707058	507	6.2	2	1	144	−8.15	8.9
26	08426335	517	5.85	1	3	167	−8.02	10.83
27	08431548	487	6.4	4	2	87	−8.02	10.68
28	02083456	573	7	0	1	186	−7.9	8.3
29	08431487	468	3	0	2	76	−7.85	9.8
30	08441514	555	5.9	0	1	136	−7.73	9.3
31	09312959	511	7	1	2	168	−7.14	11.4
32	08440589	573	4.9	0	3	175	−7.05	9.8
33	08433358	527	6.2	1	1	120	−7.01	11.4
34	00702745	512	5.5	0	0	114	−7.01	10.8
35	08433372	553	4.9	0	3	167	−6.97	10.5
36	00479883	328	2.4	2	2	100	−6.84	8.5
37	02285257	403	3	0	1	155	−6.78	10.3
38	08441298	543	5.3	0	0	154	−6.69	10.7
39	08430222	527	2.7	0	0	149	−6.37	9.7
40	00702687	523	5.8	0	0	106	−6.27	9.7
41	00929956	464	2.6	0	2	134	−6.12	9.2
42	08426332	461	3.7	0	3	127	−5.8	9.9
43	08441428	467	3.3	0	0	254	−5.53	8.8
44	02184739	374	2.8	0	0	96	−4.98	8.9
45	03354661	308	1.83	4	3	137	−6.01	8.09

a Abbreviation used for features: MW, molecule weight; xLogP, partition coefficient; PSA, polar surface area; HBA, hydrogen bond acceptor; HBD, hydrogen bond donor;

b Binding energy and score calculated by AutoDock4 and Surflex, respectively; AutoDock docking energy is in kcal/mol.

**Table 2:** Relative difference of binding (free) energy (kJ/mol, Δ*G*_bind_) resulting from molecular mechanics/Poisson–Boltzmann surface area analysis for the five OfHex1-inhibitor complexes.

Contribution	ZINC08440649		ZINC08440888		ZINC02107266		ZINC08440020		ZINC00997513	

	Mean	Std	Mean	Std	Mean	Std	Mean	Std	Mean	Std
**Δ*E*_ele_**	−22.31	7.86	−42.52	8.21	−5.99	3.44	−28.59	3.77	−20.28	5.51
**Δ*G*_vdw_**	−40.84	6.08	−27.99	2.95	−41.12	2.61	−32.56	2.33	−31.81	4.30
**Δ*G*_np_**	−7.44	0.68	−6.02	0.50	−5.87	0.25	−6.03	0.22	−5.43	0.45
**Δ*G*_PB_**	30.44	6.62	32.31	4.54	17.37	2.23	26.67	2.10	10.49	2.08
**Δ*G*_cavity_**	−7.44	0.68	−6.02	0.50	−5.87	0.25	−6.03	0.22	−5.43	0.45
**Δ*G*_gas_**	−63.16	11.76	−70.52	8.20	−47.11	4.35	−61.14	4.02	−52.09	8.02
**Δ*G*_sol_**	23.00	6.07	26.29	4.45	11.50	2.16	20.63	2.02	5.06	1.77
−***T*Δ*S***	19.62	3.2	15.86	6.3	16.89	6.9	21.17	6.4	27.80	6.5
**Δ*G*_bind a_**	−40.17	6.29	−44.23	4.54	−35.61	3.57	−40.51	3.53	−47.03	6.61
**Δ*G*_bind b_**	−20.55	-	−28.37	-	−16.92	-	−19.34	-	−19.23	-

The components of the total binding energy are also shown (kJ/mol): electrostatic (Δ*E*_ele_), van der Waals (Δ*G*_vdw_), nonpolar solvation (Δ*E*_np_), polar solvation (Δ*E*_PB_ ), gas (Δ*G*_gas_ = Δ*G*_vdw_ + Δ*E*_ele_), solvation (Δ*G*_sol_ = Δ*G*_np_ + Δ*G*_PB_), (Δ*G*_bind_^a^). The entropy term was not included in the total binding free energy. (Δ*G*_bind_^b^). The entropy term was included in the total binding free energy.
